# Thiol-disulfide exchange between the PDI family of oxidoreductases negates the requirement for an oxidase or reductase for each enzyme

**DOI:** 10.1042/BJ20141423

**Published:** 2015-07-06

**Authors:** Ojore B.V. Oka, Hui Y. Yeoh, Neil J. Bulleid

**Affiliations:** * The Institute of Molecular, Cell and Systems Biology, CMVLS, University of Glasgow, Glasgow, G12 8QQ, U.K.

**Keywords:** disulfide formation, electron transfer, endoplasmic reticulum, protein disulfide isomerase, redox potential, thiol-disulfide exchange

## Abstract

The formation of disulfides in proteins entering the secretory pathway is catalysed by the protein disulfide isomerase (PDI) family of enzymes. These enzymes catalyse the introduction, reduction and isomerization of disulfides. To function continuously they require an oxidase to reform the disulfide at their active site. To determine how each family member can be recycled to catalyse disulfide exchange, we have studied whether disulfides are transferred between individual PDI family members. We studied disulfide exchange either between purified proteins or by identifying mixed disulfide formation within cells grown in culture. We show that disulfide exchange occurs efficiently and reversibly between specific PDIs. These results have allowed us to define a hierarchy for members of the PDI family, in terms of ability to act as electron acceptors or donors during thiol-disulfide exchange reactions and indicate that there is no kinetic barrier to the exchange of disulfides between several PDI proteins. Such promiscuous disulfide exchange negates the necessity for each enzyme to be oxidized by Ero1 (ER oxidoreductin 1) or reduced by a reductive system. The lack of kinetic separation of the oxidative and reductive pathways in mammalian cells contrasts sharply with the equivalent systems for native disulfide formation within the bacterial periplasm.

## Abstract (precis)

The PDI family form disulfide bridges in substrates via thiol-disulfide exchange reactions. We show in the present study that disulfide exchange can occur directly between individual PDI proteins. Implication is that only certain members need to be oxidized or reduced to maintain function.

## INTRODUCTION

**Figure 1:**
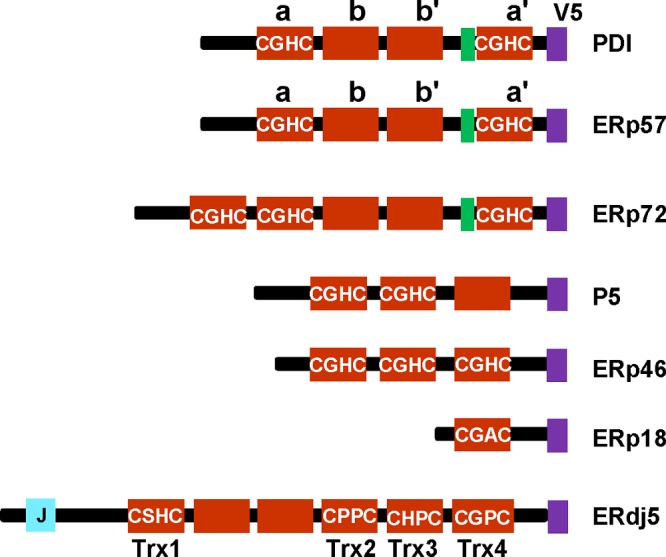
Schematic diagram showing the various PDI proteins in the present study The schematic depicts the domain organizations of various PDI proteins. The active sites are shown to indicate the variation in amino acid sequence between the cysteine residues. The domains a, b, b' and a' for PDI and ERp57 are indicated as well as the four redox active thioredoxin domains and J domain of ERdj5.

A family of proteins, collectively termed the protein disulfide isomerases (PDIs), is responsible for formation of correct disulfides in proteins entering the secretory pathway. This protein family is characterized by the presence of one or more domains structurally related to thioredoxin (Figure 1). Most members of the family contain a redox active CXXC motif; exceptions include endoplasmic reticulum (ER)p27 and ERp29 [1,2]. The relative orientation of the thioredoxin domains and their active site chemistries vary depending on the particular enzyme and may determine the specific functions of each protein [3,4].

As oxidoreductases, PDI proteins typically catalyse thiol-disulfide exchange reactions. Consequently, whether these enzymes function in the formation or breaking of a disulfide depends on the reduction potential of their CXXC active site disulfide, their ability to be recycled efficiently and their functional interaction with their substrate proteins [5,6]. For efficient introduction of disulfide bonds into substrates a robust pathway of oxidation of the PDI disulfide must exist. To reduce disulfides the active site disulfide needs to be itself reduced. These requirements emphasize the need for machineries within the ER to cater for both oxidation and reduction in PDI protein active sites.

A number of processes that oxidize PDI proteins have been characterized which require enzymes such as Ero1 (ER oxidoreductin 1), PrxIV (peroxiredoxin IV), glutathione peroxidase (Gpx^7/8^) and VKOR (vitamin K epoxide reductase) [7–10]. The Ero1 pathway is the most conserved and generally accepted to be the predominant oxidative pathway. Mammals contain two isoforms of Ero1 (Ero1α and Ero1β), which utilize the oxidizing power of molecular oxygen to generate a disulfide bond, producing hydrogen peroxide as a by-product [11–13]. The Ero1 enzymes preferentially oxidize active site thiols within the archetypal PDI with lesser activity towards ERp46 and little activity towards other PDI proteins [14,15]. The PrxIV pathway also shows selectivity towards specific PDI proteins. Most notably preference exists towards P5 and ERp46 with other PDI proteins being poorly oxidized [16]. Although no direct evidence for VKOR substrate specificity exists, it is known that only ERp18 and some of the thioredoxin-like transmembrane (TMX), membrane-bound PDI proteins, seem to be able to form mixed disulfides with VKOR [17]. Mixed disulfide formation indicates that these PDI proteins are able to reduce a disulfide within VKOR suggesting that they may also be selectively oxidized. The selectivity of these oxidative pathways suggests that a mechanism must exist to oxidize the other PDI proteins that partake in the introduction of disulfides into client proteins.

Whereas substantial progress has been made to understand how PDI proteins are oxidized, no enzyme-catalysed pathway for their reduction has been characterized to date. The necessity to reduce disulfides, both non-native and native, by PDI proteins is crucial during folding of complex disulfide-containing polypeptides, during ER-associated degradation (ERAD) and in the regulation of the unfolded protein response (UPR) [18–20]. The consequence of this reduction is that the PDI proteins will be oxidized requiring a reductive pathway to recycle the enzyme for further activity. One particular PDI protein, ERdj5, is required for reduction in disulfides prior to ERAD or correct folding [18,19]. Previously, it was suggested that cytosol-derived glutathione could be the reductant [21,22] required to recycle selected PDI proteins with reducing activity. Depletion of glutathione increases the formation of non-native disulfides in proteins [23]; however, the rate of formation of native disulfides appeared unaffected [23,24], suggesting that glutathione is dispensable for native disulfide formation. This suggests the existence of a yet to be identified pathway for selective reduction in PDI proteins such as ERdj5.

In an attempt to extend our understanding of how individual PDI family members are oxidized or reduced, we have examined the ability of various PDI proteins to exchange disulfides with one another. Using enzyme assays with purified, recombinant PDI proteins, we demonstrate that several PDI proteins can interact and exchange disulfides. In addition, the formation of mixed disulfide complexes in stable cell lines reveals that PDI proteins can react with each other within a cellular context. These results suggest the existence of a disulfide exchange cascade that transfers oxidizing equivalents between individual PDI proteins. Such ready transfer of disulfides between individual PDI proteins contrasts with the situation within the bacterial periplasm where the oxidative and reductive pathways are kinetically separated with little or no cross-talk between the disulfide exchange proteins involved in disulfide formation or reduction [25].

## EXPERIMENTAL

### Antibodies and reagents

The following mouse monoclonal antibodies were used: anti-V5 (Invitrogen), anti-V5-conjugated agarose beads (Sigma–Aldrich), anti-His (GE Healthcare) and anti-GAPDH (glyceraldehyde-3-phosphate dehydrogenase; Ambion). The rabbit polyclonal antisera raised against P5, PDI and ERp57 have been described previously [26]. *N*-ethylmaleimide (NEM) and 4-acetamido-4′-maleimidylstilbene-2,2′-disulfonic acid (AMS) were purchased from Sigma and Life technologies respectively.

### Cell culture

HT1080 cell lines were maintained in Dulbecco's modified Eagle's medium (DMEM) supplemented with 4.5 g/l glucose, 10% FBS and 0.56 mM L-glutamine. Stable cell lines expressing the substrate-trap mutants (CXXA) of the PDI oxidoreductases have been described previously [18,26].

### Recombinant protein expression and purification

The expression and purification of thioredoxin, wild-type (wt) PDI, P5 and ERp57 have been described previously [8,27]. PDI ΔS1S2 (a gift from Professor Lloyd Ruddock, Oulu Finland), in which both active sites are mutated to AGHA, was expressed and purified as described for the wt protein. Both ERp72 and ERp46 were expressed as GST-fusion proteins (pGEX-6P-1) as described [28] and purified on a 5 ml of GSTrap HP column (GE Healthcare) pre-equilibrated with 50 mM Tris/HCl, pH 7.4, buffer containing 150 mM NaCl, 1 mM EDTA and 1 mM DTT. GST-tagged proteins were eluted by the application of a linear gradient of 0–10 mM GSH (over five column volumes at a flow rate of 2 ml/min) in 50 mM Tris/HCl, pH 7.4, buffer containing 150 mM NaCl, 1 mM EDTA, 1 mM DTT and 10 mM GSH. PreScission Protease (Amersham Pharmacia) digestion of the GST-tagged recombinant proteins was carried out according to the manufacturer's protocol, to obtain ERp72 and ERp46. Isolated ERp72 and ERp46 were further purified by gel filtration using a 120 ml of Superdex 75 16/60 prep-grade column (Amersham Pharmacia) on an AKTA purifier system (GE Healthcare), in 50 mM Tris/HCl, pH 7.5, buffer containing 150 mM NaCl and1 mM EDTA.

### Reduction and oxidation of PDI proteins

PDI proteins were reduced with 10 mM DTT for 10 min at room temperature. DTT was removed using a 5 ml of Hitrap desalt column (GE Healthcare) pre-equilibrated with 50 mM Tris/HCl, pH 7.5, buffer containing 150 mM NaCl and 1 mM EDTA (buffer A), according to the manufacturer's protocol. To oxidize the proteins, samples were incubated with 10 mM GSSG for 10 min at room temperature. GSSG was removed by gel filtration using a Superdex 10/300 GL column (GE Healthcare) pre-equilibrated with buffer A. The redox status of all PDI proteins was verified by carrying out AMS alkylation and separation by SDS/PAGE.

### Gel-based thiol-disulfide exchange reaction

To determine if the PDI protein can exchange disulfides, equimolar amounts (typically 2 μM) of reduced and oxidized recombinant PDI oxidoreductases were incubated in buffer A with samples taken at specific time points. The reactions were stopped by boiling in SDS sample buffer (200 mM Tris/HCl buffer, pH 6.8, 3% SDS, 10% glycerol, 1 mM EDTA and 0.004% Bromophenol Blue) supplemented with 10 mM AMS to freeze the redox states. Finally samples were analysed under non-reducing SDS/PAGE followed by Coomassie staining or Western blotting to visualize the redox states of the individual PDI proteins. Quantification of band intensities was carried out using ImageJ software.

### Immunoisolation and Western blots

Confluent HT1080 cells and HT1080 cells stably overexpressing substrate-trapping PDI oxidoreductases were rinsed twice with PBS supplemented with 20 mM NEM. Cells were lysed in buffer containing 50 mM Tris/HCl, pH 7.4, 1% (v/v) Triton X-100, 150 mM NaCl, 2 mM EDTA and 0.5 mM PMSF (lysis buffer) supplemented with protease inhibitor cocktail (Roche). Immunoisolation and SDS/PAGE were performed as described above. For Western blotting, proteins were transferred to nitrocellulose membranes (LI-COR Biosciences), which were blocked in either 5% (w/v) dried milk or 3% BSA (for ERp57 blots) in 10 mM Tris/HCl buffer, pH 7.5, containing 150 mM NaCl and 0.1% (v/v) Tween 20 for 1 h. Blots were incubated with primary antibody for 1 h at room temperature. LI-COR IRDye fluorescent secondary antibodies were used for detection at a 1:10000 dilution. Blots were scanned using an Odyssey Sa Imaging System (LI-COR Biosciences).

### Determination of *in cellulo* redox status of ER-localized thioredoxin and ERdj5

Hela cells stably expressing doxycycline inducible human thioredoxin, engineered with a V5-tag and KDEL ER-retrieval sequence at the C-terminus, were generated using the Flp-In T-Rex Core Kit (Invitrogen). The HT1080 cell line stably overexpressing wt ERdj5 has been described previously [18]. The *in cellulo* redox state of thioredoxin and ERdj5 with AMS were determined as described previously [22].

## RESULTS

### Quantitative gel-based assay for disulfide exchange between PDI proteins

**Figure 2:**
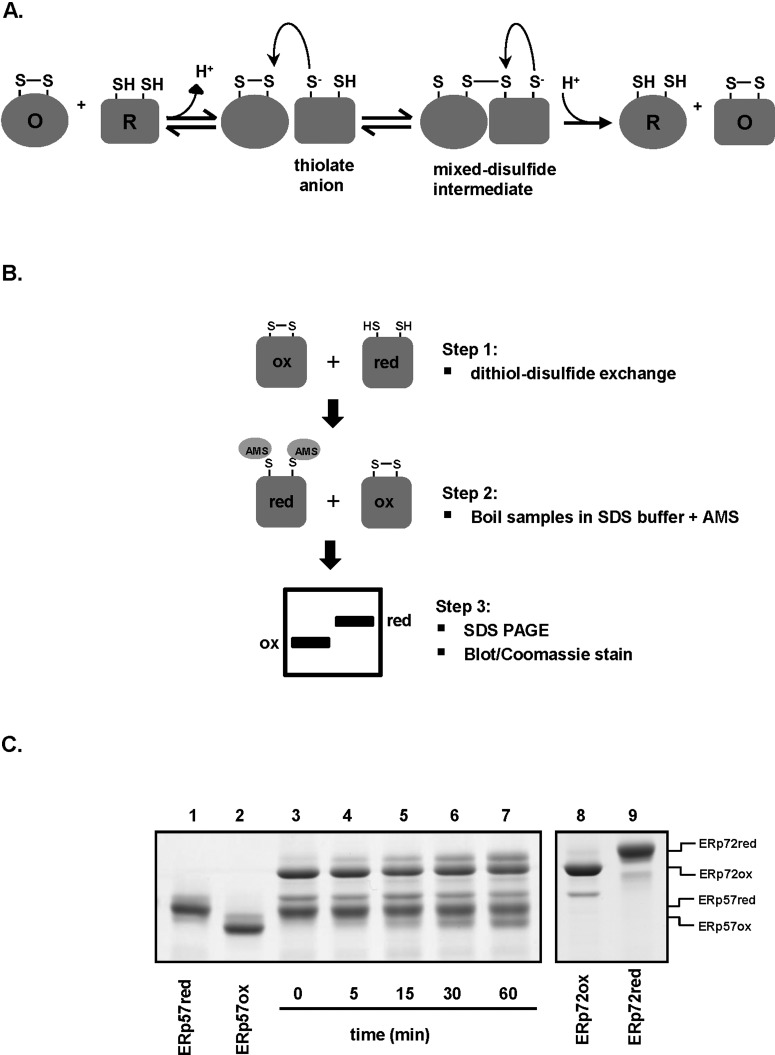
Thiol-disulfide exchange reaction between ERp72 and ERp57 (**A**) Reversible thiol-disulfide exchange between PDI proteins and their substrates occurs through a mixed disulfide intermediate. This intermediate becomes resolved by the remaining thiolate in the active site of the PDI if the overall reaction is the reduction in the substrate disulfide or by a second cysteine in the substrate if the exchange takes place in the reverse direction. The net result is the exchange of a disulfide between PDI and substrate protein. O, oxidized; R, reduced. (**B**) Schematic figure depicting the gel-shift AMS-modification enzyme assay to investigate disulfide exchange. Stoichiometric amounts (2 μM each) of reduced and oxidized recombinant PDI proteins were incubated in an endpoint time-course (step 1) for various times, samples were boiled in SDS/AMS buffer to stop reaction (step 2). Samples were then separated on non-reducing SDS/PAGE and visualized by Coomassie staining or Western blotting (step 3). (**C**) Coomassie stained gel showing disulfide exchange between reduced ERp57 and oxidized ERp72. The ERp57red, ERp72red and ERp57ox, ERp72ox indicate reduced and oxidized controls respectively.

Disulfide bond formation catalysed by the PDI proteins typically involves a bimolecular nucleophilic substitution reaction (S_N_2) initiated by the attack on a disulfide bond by a nucleophilic thiolate anion. The reaction proceeds via a transient mixed-disulfide intermediate which becomes resolved, resulting in the reduced reactant becoming oxidized and the oxidized reactant becoming reduced (Figure 2A). Such a reaction requires the participating sulfur atoms to be in a linear orientation. To determine if the active site thiols of the PDI proteins can exchange disulfides with one another, we assayed their redox status following co-incubation, by modification of free thiols with the alkylating agent AMS. Alkylation by AMS results in a mass increase of approximately 510 Da per modified thiol causing a decrease in mobility of reduced proteins relative to oxidized proteins on SDS/PAGE (Figure 2B). We elected to study ERp57 and ERp72 as substrates in these experiments as both proteins produced the most discernible gel shifts between the oxidized and reduced species, which allowed for easy quantification and interpretation of the results (Figure 2C). Stoichiometric amounts of reduced ERp57 and oxidized ERp72 were incubated together and their redox status ascertained by AMS modification at various time points. At the start of the reaction ERp57 and ERp72 were completely reduced and oxidized respectively (lane 3). However, after 5 min of incubation (lane 4), we see the appearance of a slow-migrating form of ERp72, consistent with AMS modification and reduction. The intensity of this slow-migrating ERp72 increases as time progresses (lanes 5–7). Conversely, the reduced ERp57 becomes oxidized by ERp72 in a time-dependent manner, typified by the appearance of a fast-migrating, non-AMS modified form of the protein. This result demonstrates disulfide exchange between the two proteins and suggests that the active site thiols are able to adopt the correct geometry to carry out the S_N_2 reaction. The disulfide exchange is most likely to occur via the catalytic disulfides as ERp72 does not contain any non-catalytic disulfides, the non-catalytic disulfide in ERp57 is structural and buried in the structure and we showed using a differential alkylation approach followed by MS that the non-catalytic disulfide in ERp57 is not reduced following treatment with 10 mM DTT demonstrating it is solvent inaccessible (results not shown).

### PDI proteins form a hierarchy for disulfide exchange

**Figure 3:**
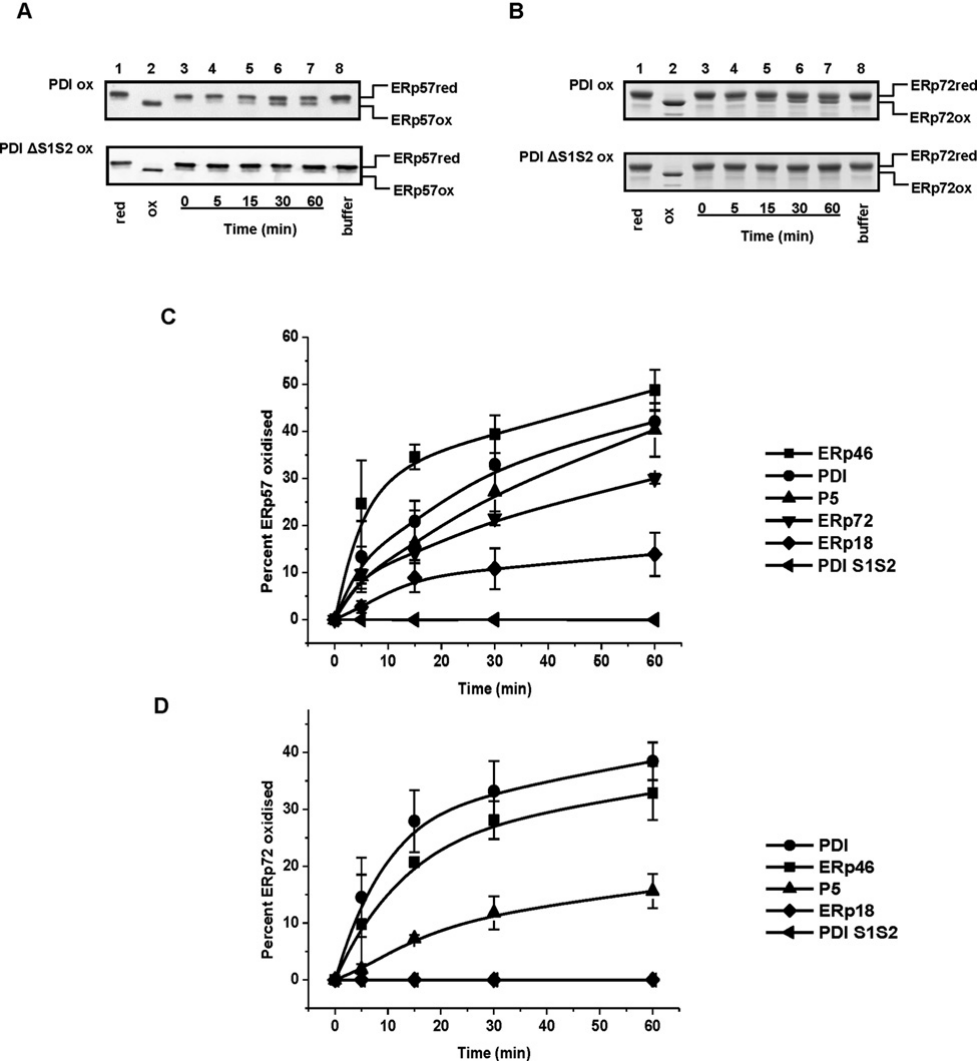
Oxidation of ERp57 and ERp72 by various PDI proteins Disulfide exchange reactions between oxidized PDIs and reduced forms of ERp57 and ERp72 were carried out as described in Figure 2. (**A**) Representative blots showing the oxidation of reduced ERp57 by PDI wt (top panel) and lack of oxidation by the active sites mutant, PDI ΔS1S2 (bottom panel). Lane 8 is ERp57 incubated alone in buffer for 60 min. (**B**) Representative Coomassie stained gels showing oxidation of ERp72 by wt PDI but not by active-sites mutant PDI or by dissolved oxygen in the assay buffer (lanes 8). Lane 1 and 2 contain reduced (red) and oxidized (ox) protein used as controls. The percentages of ERp57 and ERp72 oxidized by the various PDIs was plotted as a function of time and shown in (**C**) and (**D**) respectively. The error bars represent±S.D. for at least three independent experiments.

Having established that reduced ERp57 and oxidized ERp72 engage in disulfide exchange, we then examined if other PDI proteins can exchange disulfides with ERp57 and ERp72. To determine if various oxidized PDIs can accept electrons from reduced forms of ERp57 and ERp72, stoichiometric amounts of oxidized PDI proteins including ERp46, ERp18, P5, ERp72, PDI wt and PDI ΔS1S2 (both active sites mutated to AGHA) were incubated with reduced ERp57 and ERp72 (Figure 3). We included a PDI ΔS1S2 mutant in these assays as a control to demonstrate that any disulfide exchange observed with the wt PDI could only be due to its active site disulfide. Representative results from the incubation of either oxidized PDI or oxidized PDI ΔS1S2 with reduced ERp57 are shown (Figure 3A). Quantitative Western blotting was carried out rather than Coomassie Blue staining as PDI has a similar mobility to ERp57. Reduced and oxidized ERp57 controls are indicated in lanes 1 and 2 respectively. As observed with ERp72 (Figure 2C), after 5 min of incubation with PDI, a fast-migrating form of ERp57 appeared which increased in intensity up to 60 min of incubation (lanes 3–7). This is consistent with the oxidation of ERp57 by PDI. In contrast, reduced ERp57 incubated in assay buffer only for the entire duration of the time course (lane 8) or with oxidized PDI ΔS1S2 (Figure 3A, bottom panel), was not oxidized, as demonstrated by the absence of the fast-migrating form of the protein. These results demonstrate that the oxidation of ERp57 is catalysed by PDI and that disulfide exchange requires the PDI active sites disulfides. Similar results were obtained when PDI was incubated with ERp72 (Figure 3B). The result reveals a faster-migrating form of ERp72, only evident upon incubation with wt PDI (top panel) but not with the active sites mutant, PDI ΔS1S2 (bottom panel).

The ability of other PDI proteins to exchange disulfides with ERp57 and ERp72 was determined and the time courses of oxidation quantified (Figures 3C and 3D). The results suggest that the various PDI proteins exchange disulfides with ERp57 and ERp72 to varying degrees. It should be noted that the ability of each PDI to exchange disulfides will be dependent upon several factors potentially including the number of catalytically active disulfides in the donor and recipient (Figure 1). For example, the oxidized ERp46 (three disulfides) was most efficient in exchanging disulfides with reduced ERp57 (two disulfides; Figure 3C) whereas ERp18 (1 disulfide) was relatively inefficient (Figure 3C). Perhaps a better comparison is between PDI proteins that have the same number of active site disulfides such as PDI and P5. For ERp72, PDI oxidized the recipient active site disulfides more rapidly and to a greater extent than P5 (Figures 3C and 3D). Strikingly, ERp18 only slowly exchanged disulfides with ERp57 and was unable to exchange disulfides with reduced ERp72 (Figure 3D). Our results do not allow us to define which of the thioredoxin domains within individual PDI proteins becomes reduced or oxidized; however, they do allow us to conclude that disulfide exchange is occurring. Taken together these results show that there is a hierarchy of ability of PDI proteins able to exchange active site disulfides, with PDI and ERp46 being the most and ERp18 the least efficient.

**Figure 4:**
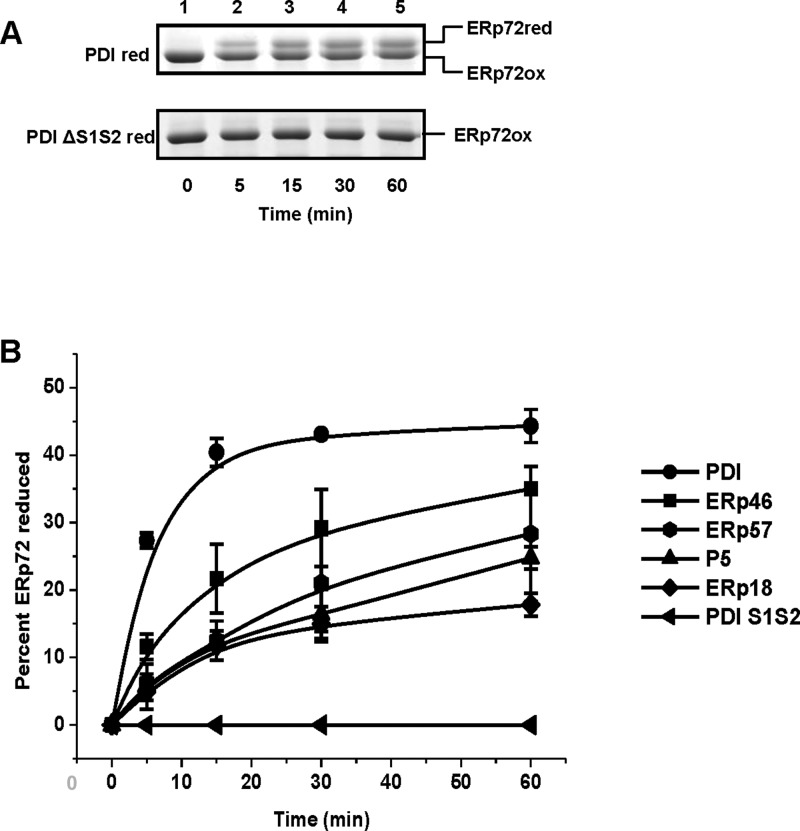
Reduction in oxidized ERp72 by various PDI proteins Stoichiometric amounts of reduced PDIs and oxidized ERp72 were assayed as described in Figure 2. (**A**) Representative Coomassie stained gels showing the reduction in oxidized ERp72 by PDI. Wt (top panel); PDI ΔS1S2 (bottom panel). (**B**) The percentages of reduction in ERp72 by the various PDI proteins were plotted against time. The error bars represent±S.D. for at least three independent experiments.

Having established that various oxidized PDIs can exchange disulfides with reduced forms of ERp57 and ERp72, we next examined the possibility of electron transfer between reduced PDI proteins and oxidized ERp72. An equivalent experiment with ERp57 could not be performed due to the insolubility of the oxidized protein. Stoichiometric amounts of reduced PDIs including ERp46, ERp18, P5, ERp57, PDI and PDI ΔS1S2 were incubated with oxidized ERp72 (Figure 4). A representative time course with reduced PDI and reduced PDI ΔS1S2 (Figure 4A) demonstrates the reduction in ERp72 by reduced PDI but not the active-site mutant protein. The results obtained from assays involving oxidized ERp72 and reduced forms of PDI, ERp46, ERp18, P5 and ERp57 indicate that several PDIs, to varying degrees, can exchange disulfides with oxidized ERp72 (Figure 4B). PDI is the most efficient exchanger of disulfides with reduced ERp72 with equilibrium being reached within 20 min. We also noted that reduced ERp18 can transfer electrons to oxidized ERp72 contrasting with the lack of disulfide exchange between oxidized ERp18 and reduced ERp72.

### Thioredoxin can efficiently donate electrons to PDI proteins

**Figure 5:**
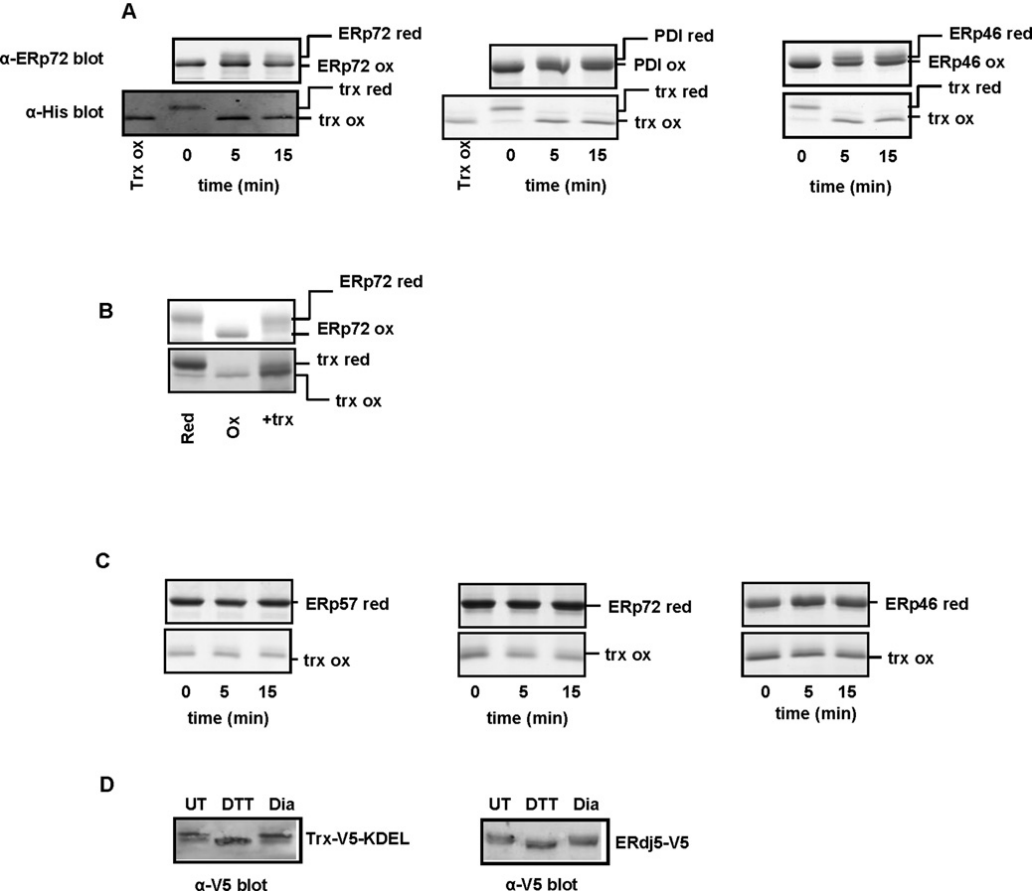
Reduced thioredoxin rapidly exchanges disulfides with oxidized PDI proteins His-tagged thioredoxin and various PDI proteins were incubated together and the extent of disulfide exchange assayed as described in Figure 2. (**A**) Reduced thioredoxin can efficiently transfer electrons to oxidized PDI proteins. Left panel shows representative Western blots from reaction between stoichiometric amounts of ERp72 and thioredoxin; α-ERp72 (top) and α-His (bottom). The middle and right panels include Coomassie stained gels for reactions involving reduced thioredoxin and oxidized PDI and ERp46 respectively. (**B**) Reduction in ERp72 when incubated with an excess of thioredoxin (**C**) Representative Coomassie stained gels indicating no disulfide exchange when reduced PDI proteins are incubated with oxidized thioredoxin. Left, middle and right panels indicate reactions between thioredoxin and ERp57, ERp72 and ERp46 respectively. (**D**) ER-localized thioredoxin and ERdj5 are predominantly oxidized at steady state. Hela cells stably expressing doxycycline-inducible ER-localized V5-tagged thioredoxin and HT1080 cells overexpressing V5-tagged ERdj5 were either left untreated (UT) or treated with DTT (10 mM) or diamide (Dia; 5 mM) for 5 min. The samples were subjected to modification with AMS, followed by SDS/PAGE and anti-V5 blot.

We have shown that various ER-localized PDIs, with similar active site chemistries and redox potentials can engage in disulfide exchange with varying efficiencies resulting in mixtures of partially reduced and oxidized proteins. In particular, our results show that PDI and ERp72 can engage in reversible disulfide exchange; it did not matter which protein was reduced or oxidized, disulfide exchange was still relatively efficient. In order to examine to what extent the direction of disulfide exchange can be driven by the reduction potential of the active-site disulfide, we assayed the ability of thioredoxin, a cytosolic protein with a comparatively low redox potential (−270 mV) [29] to exchange disulfides with various PDI proteins. Ideally we would have used ERdj5 for these experiments as it has the lowest reduction potential of the PDI family [30] but unfortunately we were unable to produce sufficient recombinant protein. When reduced thioredoxin was incubated with a stoichiometric amount of oxidized ERp72, PDI or ERp46 disulfide exchange was very efficient (Figure 5A). Within 5 min of incubation, the thioredoxin was completely oxidized, with corresponding reduction in the PDI proteins. When included in excess, reduced thioredoxin completely reduced ERp72 (Figure 5B). On the other hand, no disulfide exchange occurred when oxidized thioredoxin was incubated with reduced ERp57, ERp72 or ERp46 (Figure 5C). This result demonstrates that the extent of disulfide exchange is determined primarily by the reduction potential of the active-site disulfide.

The ability of thioredoxin to efficiently reduce the PDI proteins provided us with the opportunity to evaluate how efficient the reductive pathway is within the ER at recycling oxidized thioredoxin domains with low reduction potentials. Within the bacterial periplasm, DsbC is maintained primarily in the reduced state [31] by a robust reductive pathway involving the membrane protein disulfide bond (DsbD) [32]. Thioredoxin is localized to the cytosol where it is fully reduced. Hence, it would not normally interact with the PDI proteins, but when thioredoxin was targeted to the ER of mammalian cells by appending a signal sequence at the N-terminus and a KDEL retrieval sequence at the C-terminus it was fully oxidized (Figure 5D). Note that slower migrating bands in this case indicate oxidized protein as free thiols are first blocked with NEM and disulfides are subsequently reduced and then alkylated with AMS. In addition, we observed that the primary ER reductase ERdj5 was also fully oxidized in mammalian cells, indicating that proteins containing thioredoxin domains with low reduction potential are efficiently oxidized in the ER lumen. These results contrast with the situation in the bacterial periplasm and suggest that the reductive pathway in the ER is not kinetically separated from the oxidative pathway.

### The PDI proteins form mixed-disulfide complexes *in cellulo*

Thiol-disulfide exchange reactions typically involve the formation of a transient mixed-disulfide intermediate between enzyme and substrate, which is resolved to yield the final reaction products (Figure 2A). Previous studies have shown that mutation of the PDI CXXC active site motif to CXXA (cysteine to alanine) stabilizes the mixed-disulfide intermediate formed between the enzyme and substrate, allowing the identification of substrates for PDI proteins [9,18,26,33].

**Figure 6:**
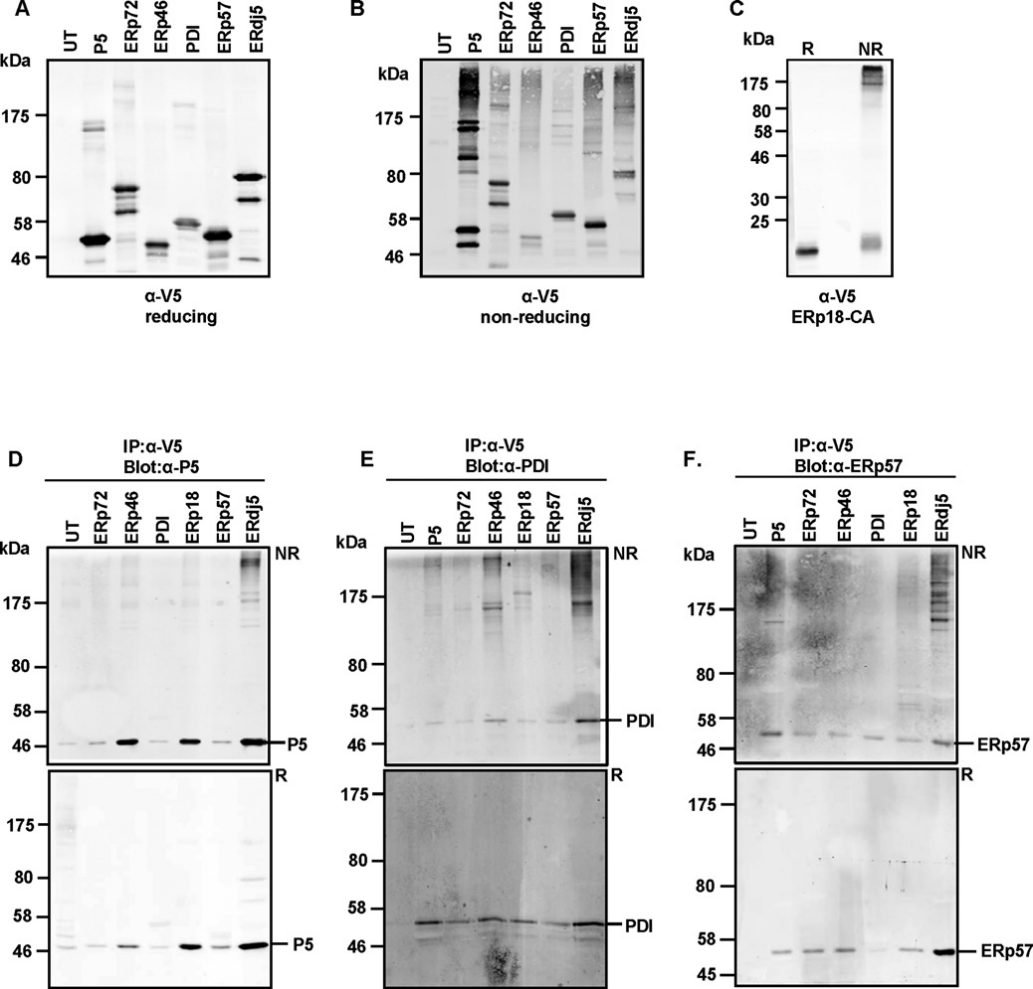
PDI oxidoreductases form mixed-disulfide complexes Cell lysates from HT1080 cells, either untransfected (UT) or stably expressing the V5-tagged substrate-trapping mutants of the PDI proteins indicated, separated under reducing (**A**) or non-reducing (**B**) conditions were immunoblotted using the V5 antibody to detect the exogenously expressed PDI proteins. Anti-V5 blot to detect exogenous ERp18 is shown in (**C**). HT1080 cells either untransfected (UT) or stably expressing the PDI substrate-trap mutants were subjected to immunoprecipitation with the V5 antibody. Immunoisolated material was separated by SDS/PAGE and analysed by immunoblotting with antibodies against P5 (**D**), PDI (**E**) or ERp57 (**F**). (R and NR indicate reducing and non-reducing SDS/PAGE conditions).

To extend these studies, and in light of our observations that various PDIs can exchange disulfides *in vitro*, we used the substrate-trap approach to investigate if members of the PDI family can form mixed-disulfides complexes with each other in cells grown in culture. HT1080 stable cell lines overexpressing V5-tagged CXXA mutants of PDI proteins were lysed in buffer containing NEM to trap any mixed disulfides. The expression of each PDI protein was verified by carrying out a V5-Western blot (Figure 6A). When the cell lysates were analysed under non-reducing SDS/PAGE, several V5-reactive high-molecular-mass complexes were identified (Figures 6B and 6C, for ERp18). These results demonstrate the presence of mixed disulfides between the various PDI proteins and endogenous client proteins.

To determine the presence of other PDI proteins in the mixed-disulfide complexes, we immunoisolated the exogenously expressed PDI protein and separated the immunoisolated material on reducing and non-reducing SDS/PAGE. Western blot analyses were then carried out using antisera raised against specific PDI proteins, including P5, PDI and ERp57 (Figures 6D–6F respectively). To confirm that the interactions were specific to the exogenously expressed PDI protein, untransfected HT1080 cell lysates were immunoisolated as well and used as control. The anti-P5 Western blots reveal extensive interactions between endogenous P5 and other PDI proteins, notably ERp46, ERp18 and ERdj5. Most of the immunoisolated P5 was not present as mixed-disulfide complexes, suggesting that P5 forms non-covalent interactions with some but not all PDI proteins. Mixed-disulfide complexes were formed between P5 and ERdj5 (Figure 6D, top panel) suggesting that disulfide exchange can occur between these two PDI family members within a functionally intact ER. It is of interest to note that P5 has been shown to reduce a disulfide in the luminal domain of inositol-requiring enzyme 1 (IRE1) and in the process regulate the UPR [20]. P5 will become oxidized in the process so the formation of mixed disulfides between P5 and ERdj5 might represent a mechanism to reduce and maintain P5 in a state competent to interact with IRE1.

Western blots were also carried out to determine interactions between the various PDI proteins and endogenous PDI (Figure 6E). The result indicates that endogenous PDI forms mixed disulfides with P5, ERp46, ERp18 and most notably ERdj5 (Figure 6E, top panel). Interestingly, we did not detect mixed disulfides between PDI and the substrate-trapping mutants of ERp57 and ERp72, despite the exchange of disulfides between these proteins in our *in vitro* assays (Figure 3A and 3B).

Western blotting of immunoisolated material using anti-ERp57 also revealed most prominently an interaction with ERdj5 (Figure 6F). The non-reducing gel blot (top panel) shows extensive mixed-disulfide complexes between ERp57 and ERdj5. The presence of multiple mixed disulfide species of ERdj5 with each of the PDI proteins tested indicates the ability to form higher order structures stabilized by interchain disulfide bonds.

## DISCUSSION

**Figure 7:**
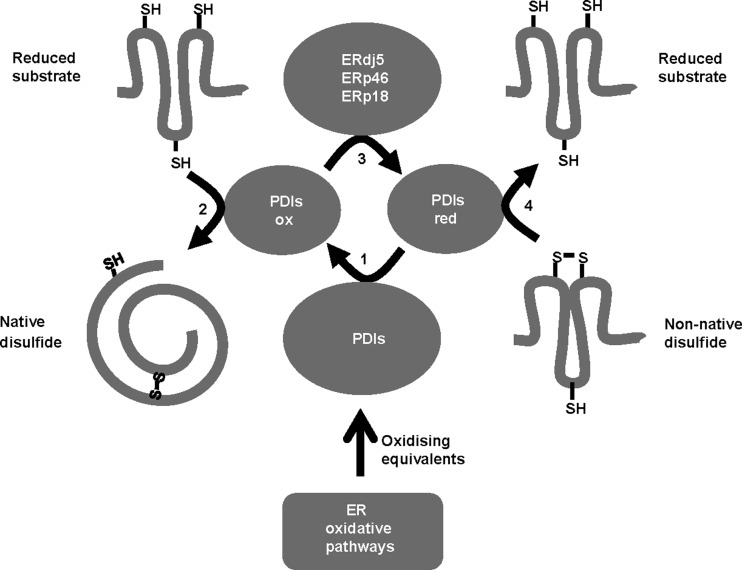
A simple model of the role of PDI oxidoreductases in oxidative folding Oxidized PDIs, in addition to components of the ER oxidative pathways, may well supply oxidizing equivalents to other PDIs (1), allowing for introduction of disulfides into newly synthesized substrates (2). Reduced PDIs can transfer electrons to other PDIs (3), allowing them to carry out reduction in non-native disulfides (4). Combination of steps 1–4, may operate to ensure formation of native disulfide bonds in proteins.

In the present study, we demonstrate the ability of various purified PDI proteins to exchange disulfides with each other and show that similar exchange reactions can occur within mammalian cells. It would appear that PDI and ERp46 are most likely to be the conduits between Ero1 and the ER disulfide exchange proteins and that ERdj5 is most likely to provide the conduit between the ER disulfide exchange proteins and any putative reduction pathway. Hence, the oxidative and reductive pathways in the ER lumen need only interface with a restricted number of PDI proteins to allow passage of electrons between most of the other members of the family (Figure 7).

The fact that exchange reactions can occur relatively efficiently raises the question of how the opposing pathways of disulfide formation and reduction are regulated within the mammalian secretory pathway. Within the bacterial periplasm, the two pathways are kinetically segregated so that although the active site reduction potentials of the disulfide exchange proteins DsbA and DsbC are similar [34,35], DsbA is primarily oxidized and catalyses the introduction of disulfides whereas DsbC is reduced and reduces non-native disulfides or oxidized thiols [36]. Such segregation of the pathways is achieved by the lack of disulfide exchange between DsbA and DsbC and the inability of DsbB and DsbD to oxidize or reduce DsbC or DsbA respectively [35]. Within the mammalian ER there does seem to be some specificity for the oxidative pathways to oxidize specific PDI proteins but as these can then exchange disulfides with each other, there is no requirement for direct recycling of each PDI protein by Ero1, PrxIV, Gpx7/8 or VKOR. Likewise, a reductive pathway would only need to reduce ERdj5 which will then go on to reduce other PDI proteins. Hence, a defined kinetic segregation of the two opposing oxidative and reductive pathways does not appear to be present within the ER of mammalian cells.

The fact that ERdj5 and ER-localized thioredoxin are mostly oxidized would also suggest that the ER reductive pathway is inefficient or tightly regulated to prevent excessive reduction in the PDI proteins and to favour disulfide formation. Such a reductive pathway may only be activated upon hyperoxidizing conditions within the ER lumen such as occurs during the UPR [37] or during calcium depletion [38]. Reduction in ERdj5 would reduce the active site disulfides in other PDI proteins effectively preventing further disulfide formation and favouring reduction in non-native disulfides to allow subsequent protein folding or to facilitate dislocation of the protein to the cytosol for degradation. Reduction in regulatory disulfides within redox sensitive ER resident proteins may also occur under such conditions providing an additional layer of regulation following the UPR or oxidative stress [20].

Whereas most of the PDI proteins tested were able to exchange disulfides, there was some selectivity both with purified proteins and with the substrate-trapping mutants expressed in cells. In particular, reduced ERp72 was unable to transfer electrons to oxidized ERp18, whereas oxidized ERp72 can be reduced by ERp18. If the reaction was solely determined by the active site chemistries then the reaction should proceed in both directions until equilibrium is reached. As their calculated redox potentials are quite similar [15,39], there must be an alternative explanation. NMR studies have shown that the oxidized and reduced states of ERp18 have chemical shift differences between residues in and around the active-site disulfide, consistent with different conformational states [40]. Hence, oxidized ERp18 may simply form a conformation that does not interact with ERp72, which prevents disulfide exchange.

The apparent absence of mixed disulfides formation between some PDI proteins tested *in cellulo* contrasts with their ability to exchange disulfides *in vitro*, for example between PDI and ERp57 or ERp72. Demonstration of disulfide exchange with purified proteins shows that this reaction can occur efficiently but it does not mean that this will occur in cells where the individual components may be in complex with other proteins precluding their interaction. For example, ERp57 is in complex with calnexin, calreticulin and also ERp27 [2]. Hence, the lack of mixed disulfide formation between PDI and ERp57 indicates that ERp57 might primarily act as a reductase and the formation of a mixed disulfide with ERdj5 supports this conclusion.

In summary our results raise some intriguing questions about how the balance of oxidation and reduction in disulfide bonds is regulated in the ER lumen and suggest the absence of a clear kinetic segregation of the pathways. Given the promiscuity of disulfide exchange, further work needs to be carried out to identify the mechanism for reduction in PDI proteins such as ERdj5 and to determine how this pathway is regulated to prevent excessive reduction in disulfides to favour disulfide formation.

## Abbreviations

AMS: 4-acetamido-4′-maleimidylstilbene-2,2′-disulfonic acid
ER: endoplasmic reticulum
Ero1: ER oxidoreductin 1
NEM: N-ethyl maleimide
PDI: protein disulfide isomerase
PrxIV: peroxiredoxin IV
VKOR: vitamin K epoxide reductase
wt: wild-type
